# Promoting mental health in the age of new digital tools: balancing challenges and opportunities of social media, chatbots, and wearables

**DOI:** 10.3389/fdgth.2025.1560580

**Published:** 2025-03-13

**Authors:** Julien Coelho, Florian Pécune, Jean-Arthur Micoulaud-Franchi, Bernard Bioulac, Pierre Philip

**Affiliations:** ^1^ University Bordeaux, SANPSY, CNRS, UMR 6033, Hôpital Pellegrin, Bordeaux, France; ^2^ Service Universitaire de Médecine du Sommeil, CHU de Bordeaux, Bordeaux, France; ^3^ University Bordeaux, IMN, CNRS, UMR 5293, Centre Broca Nouvelle-Aquitaine, Bordeaux, France

**Keywords:** digital tools, mental health, social media, chatbots, wearables

## Abstract

The promotion of mental health is essential for global health, affecting millions with disorders such as anxiety and depression. Although stigma and discrimination hinder progress, these conditions are often preventable or manageable at minimal cost. The adoption of digital tools in mental health promotion, including telemedicine, online therapy, social media, and wearables, offers promising new avenues to address these issues. This review proposes a framework that focuses on the use of digital tools to enhance health literacy, foster behavioral change, and support sustained positive health behaviors. Platforms such as TikTok, Facebook, and Instagram can effectively disseminate health information, increase awareness, and enhance social accountability. Artificial intelligence-driven virtual agents offer personalised mental health interventions, providing motivational support and customised advice. Additionally, wearable technology (e.g., fitness trackers and smartwatches) enables real-time monitoring of vital health metrics, encouraging ongoing healthy activities. Nonetheless, these technologies introduce challenges including privacy issues, data security, and equitable access to digital resources, raising a new class of rights to protect mental privacy, guard against algorithm bias, and prevent personality-changing manipulations. The absence of human interaction in fully digital solutions also raises concerns about a lack of empathy and emotional connection. For optimal use of digital tools in mental health, integration with conventional care practices and adaptation to diverse cultural and social backgrounds are necessary. The results of this review suggest that digital tools, when carefully implemented, can significantly improve mental health outcomes by making care more accessible, tailored, and effective, especially for underserved communities.

## Introduction

According to the 1986 Ottawa Charter of the World Health Organization (WHO), health promotion is defined as the process that enables people to increase control over and improve their health (1). The foundational strategies of health promotion integrate societal (e.g., governance and policies), social (e.g., community involvement), and individual elements (e.g., literacy enhancement) (2). Mental health, in particular, faces unique challenges, including stigma, discrimination, and human rights violations (3). Fortunately, many mental health conditions can be effectively prevented or treated at a relatively low cost (4).

Digital tools have become widely embedded in mental health promotion, including telemedicine, online therapy, and 24/7 availability (5). A new generation of digital tools, such as social media, chatbots, conversational agents, large language models, and wearables, introduces both new challenges and opportunities. These tools aim to advocate for factors that improve mental health, enable mental health equity for all, and mediate through collaboration across various sectors (6). However, individuals with poor mental health are particularly susceptible to the drawbacks of digital tools due to challenges such as misinformation, privacy risks, potential symptom exacerbation through negative online interactions, and limited access due to financial constraints (7).

In this comprehensive review, we propose a framework for the usability of these new digital tools to promote mental health, considering their specific benefits and drawbacks. We expect this framework to foster more optimised and reasoned use of digital tools in the field of mental health.

### Steps to promote mental health

In 2019, mental disorders affected approximately 970 million individuals worldwide; anxiety and depression were the most prevalent disorders (8). The COVID-19 health crisis has exacerbated this situation (8). Mental health conditions can significantly impact all areas of life, including relationships with family, friends, and community, and may contribute to challenges in educational and work settings (9). Globally, mental disorders are responsible for one in every six years lived with disability (10). Those with severe mental health conditions have a life expectancy 10–20 years shorter than the general population (10); they also face increased risks of suicide and human rights violations (11). Additionally, the economic impact is substantial, such that productivity losses far exceed the direct costs of treatment (12). Fortunately, many mental health conditions can be effectively prevented or managed at a relatively low cost^3^. However, the technological and economic challenges faced by manufacturers do not always align with the public health goals of mental health promotion (6).

At the individual level, mental health promotion involves the encouragement of healthy behaviors such as regular physical activity, balanced nutrition, adequate sleep, and social interaction (3). Extensive research over several decades has identified the barriers and facilitators to behavioral change (13). Common to most theories, models, and frameworks are three steps: beliefs (i.e., the evaluation of the behavior as favourable or unfavourable), intention (i.e., the motivational factors that influence behavior), and behaviors (i.e., the initiation and maintenance of the behavior) (14). For example, in addiction treatment such as smoking cessation, an individual must believe that tobacco harms their mental health, be motivated to quit, take action, and sustain the behavior change (15). Mental health promotion aims to support the individual in navigating these various states (3). Each stage of change has specific needs (14). For instance, an individual unconvinced that addiction harms their mental health needs relevant health information (16). An individual who is convinced but lacks practical cessation methods requires detailed guidance on overcoming their addiction (17). An individual in the process of quitting needs support to maintain their behavior change. Interventions not tailored to the specific needs of the individual are ineffective (14).

The advent of new digital tools provides novel opportunities in mental health promotion: (i) personalised and broadly disseminated information to promote health literacy (e.g., social media), (ii) customised guidance to encourage new healthy behaviors (e.g., chatbots), and (iii) continuous monitoring of the benefits of these behaviors to aid in their maintenance (e.g., wearables) (18). However, each tool has unique characteristics that must be carefully considered to ensure its safety and effectiveness (6). In this article we aimed to propose a framework that focuses on the use of digital tools to enhance health literacy, foster behavioral change, and support sustained positive health behaviors. To ensure a comprehensive review, we systematically selected literature from peer-reviewed sources, focusing on studies published within the last five years that assessed the efficacy and challenges of digital mental health interventions. We also incorporated expert opinions and policy reports to provide a well-rounded analysis of current trends and emerging issues in digital mental health. The following sections will detail the challenges and opportunities in designing these new digital tools.

### Promoting health beliefs

Social media can help promote health beliefs, which can initiate awareness about poor health behaviors. Social media encompasses online platforms and applications that allow users to create, share, and interact with content in real-time. Popular examples include Facebook, Instagram, X, TikTok, and LinkedIn. These platforms facilitate global connections, communication, and information sharing through text, images, videos, and links. They play an important role in shaping public opinion, marketing, news distribution, and social movements. With billions of active users, social media has revolutionised how we interact, share ideas, and access information (19). Several studies have already demonstrated the effectiveness of social network-based interventions in modifying health behaviors to promote mental health. For instance, a Facebook-based intervention aimed at reducing sedentary behavior in a population of teleworkers (20), while another successfully decreased alcohol and cannabis consumption over a three-month period among emerging adults (21).

Social media can significantly optimise contemplation for prevention and public health in various ways. It enables organisations and influencers to disseminate information about mental health, share coping strategies, and promote mental well-being (22). Campaigns such as #MentalHealthAwareness can reach large audiences, encouraging discussions that normalise mental health struggles and treatment. The sharing of personal stories and journeys also can help dismantle stigma, making it easier for others to seek help. Furthermore, social media links users to mental health resources including hotlines, therapy platforms, online support groups, and professional advice (23), thereby providing immediate assistance or referrals, especially in crises. It facilitates peer-to-peer support, creating communities for individuals facing similar mental health challenges, offering emotional support, practical advice, and a sense of belonging (24). Finally, the unique features and demographics of each social platform make them suitable for promoting contemplation and facilitating public health discussions across diverse cultures and societies (25).

Social media also presents challenges and concerns. Not all content on social media is accurate or beneficial. Unverified advice or misleading information, often driven by social trends rather than health expertise, can lead to poor mental health decisions and confusion about treatment options (26). Public discussion of mental health issues also can risk privacy violations, judgment, or discrimination (19). Regulations such as the General Data Protection Regulation in Europe and the California Consumer Privacy Act in the U.S. highlight the disparity in data protection standards. Furthermore, social media can promote unrealistic health ideals, such as never experiencing depression or the ability to engage in sports daily, encouraging costly or inappropriate behaviors (e.g., prolonged diets or expensive foods without proven benefits). This can lead to unfavourable comparisons, exacerbating anxiety or depression (26). Although online communities are valuable, they cannot replace professional care. Overreliance on social media for mental health support may delay proper treatment from qualified professionals. Additionally, negative interactions such as cyberbullying can harm the mental health of users, particularly affecting those already vulnerable or with psychiatric disorders (27).

It is crucial for these platforms to ensure that the information shared is accurate, trustworthy, and culturally sensitive. The provision of resources for further learning or seeking professional advice can aid individuals in making informed health decisions. Collaborations with public health authorities, NGOs, and health professionals can amplify these efforts and contribute to overall mental health and well-being.

### Refining the intention toward behaviors

Chatbots, which are virtual agents or large language models, can strengthen the intention to change health behaviors by addressing the intermediate stage where an individual may continue an unhealthy behavior despite knowing its consequences (14). A chatbot is a human-machine interface, often driven by artificial intelligence or a large language model, designed for interaction through text or conversational interfaces. Chatbots provide assistance, answer questions, and perform tasks efficiently and empathically (28). In mental health, chatbots facilitate motivational interviews and tailored guidelines (29). They can operate independently or under caregiver supervision, aligning with proactive mental health care (PHMC) models (30). Several studies have already demonstrated the effectiveness of chatbots interventions in modifying health behaviors to promote mental health. For instance, a chatbot-based cognitive behavioral therapy aimed at reducing depressive symptoms among 49 young adults (31), while another study suggests that a motivational interview delivered by a chatbot can help motivate smokers to quit (32).

Chatbots offer numerous opportunities to promote health behaviors. They provide round-the-clock support, offering immediate assistance to individuals in need, regardless of time or location (33). The anonymity and nonjudgmental nature of chatbots may also encourage users to disclose sensitive information more freely, which facilitates open communication and help-seeking behavior (33). Moreover, chatbots can simultaneously reach a wide audience, making mental health support more accessible to large populations, including those in remote or underserved areas (33). Chatbots also complement social media in promoting good health beliefs (34).

Through machine learning algorithms, chatbots tailor interactions and recommendations to individual needs, preferences, and progress, enhancing intervention effectiveness (33). They also work in conjunction with wearables to maintain good health behaviors (34). Furthermore, chatbots can potentially reduce mental health care costs by automating routine tasks, thereby freeing up resources for more specialised interventions and reducing healthcare system burdens (33). Finally, they collect valuable data regarding user interactions, preferences, and outcomes, which can inform research, improve understanding of mental health issues, and enhance intervention development (33).

Although chatbots hold promise in the promotion of mental health, several potential issues need consideration. Despite advancements in virtual agent technology, chatbots may lack the empathetic understanding and emotional connection that human interactions provide, which could leave users, particularly those with psychiatric disorders, feeling isolated or misunderstood. Additionally, privacy concerns may deter users from disclosing sensitive mental health information due to fears of breaches or unauthorised data access. Access to the necessary technology to interact with chatbots might not be available to all, potentially worsening disparities in mental health support. Furthermore, there is a risk that chatbots will provide incorrect or harmful information without the oversight of a health professional, raising concerns about liability in the event of errors (29).

It is important for chatbots to ensure the information they provide is accurate, trustworthy, and culturally sensitive. Integration of these tools with existing healthcare structures may improve their effectiveness.

### Promoting and maintaining health behaviors with a 24/7 ecological setup

Wearables are compact, technology-enabled tools worn by individuals to monitor and collect health data in real time, such as health monitoring watches, bracelets, rings, and glasses (35). They can promote and maintain health behaviors, representing the final stage where an individual continues or discontinues a health behavior that has already been initiated (14). These devices can be valuable tools for the maintenance of healthy behaviors by providing continuous feedback, reliable and valid data, and personalised insights, thus empowering individuals to make informed health decisions (35). Several studies have already demonstrated the effectiveness of wearable-based interventions in modifying health behaviors to promote mental health. For instance, the “superpower glass,” worn by 71 children with autism spectrum disorder, aimed to improve social behavior (36), while the use of a Fitbit combined with lifestyle physical activity successfully reduced alcohol consumption and improved mental health over a 12-week period among women with alcohol use disorder (37).

Wearables offer numerous opportunities to maintain mental health behaviors. They provide real-time monitoring of various health metrics including physical activity, heart rate, sleep patterns, and stress levels. Tracking these parameters helps individuals gain a better understanding of their health status and make necessary adjustments. For example, if a wearable detects irregular sleep patterns, it can prompt the user to adopt better sleep hygiene practices (38). Immediate feedback encourages a proactive approach to health, motivating users to maintain positive behaviors (38).

Users benefit from personalised health insights and recommendations through data analysis and machine learning algorithms (39). These tailored suggestions are based on an individual's unique health data, preferences, and goals. For example, if a decline in physical activity is detected, the device might suggest specific exercises or daily activities to motivate the user (39). Personalisation enhances the relevance and effectiveness of health interventions, making it easier for individuals to adhere to their routines (40).

Wearables also incorporate goal-setting features and gamification elements to make the maintenance of health behaviors engaging and motivating. Users can set daily, weekly, or monthly health goals and track their progress through visual dashboards and achievement badges (41). Gamification, such as earning rewards for meeting activity targets or participating in challenges, adds an element of fun and competition, boosting adherence to health behaviors (41).

These devices can detect early signs of potential health issues, prompting timely interventions (41). For example, a wearable with an actigraphy feature can detect insufficient or irregular sleep and alert the user to seek medical attention (41). Early detection of health anomalies enables proactive measures, preventing the escalation of health problems and supporting overall mental well-being (41).

Although wearables offer numerous benefits, several challenges must be addressed to ensure their effective use in the promotion and maintenance of health behaviors. Privacy and data security are paramount because these devices collect sensitive health information. Users need assurance that their data are protected and used responsibly. Additionally, data protection regulations substantially vary among countries, such as the General Data Protection Regulation in European countries vs. U.S. regulations; not all countries have sovereignty over data collected in their territory (42).

Accessibility and affordability also present concerns because some individuals may not have access to the latest wearable technology. Mechanisms that ensure inclusivity and equitable access to these tools are essential for a broad-reaching public health impact (43). Additionally, the constant production of ecological data can encourage pathological behaviors such as orthorexia or promote an unhealthy hyperfocus on health behaviors in some patients, potentially leading to harmful effects (44).

Another challenge is that biomarkers from wearables are usually measured indirectly, often computed from algorithms or artificial intelligence systems that vary among tools. Their reliability and validity represent a challenge, especially because very few are registered following FDA/CE medical device standards (45).

In all cases, it is essential for wearables to ensure that the information provided is accurate, trustworthy, and culturally sensitive. Additionally, the integration of these tools with an existing healthcare structure may increase their effectiveness.

## Conclusion

**Figure 1:**
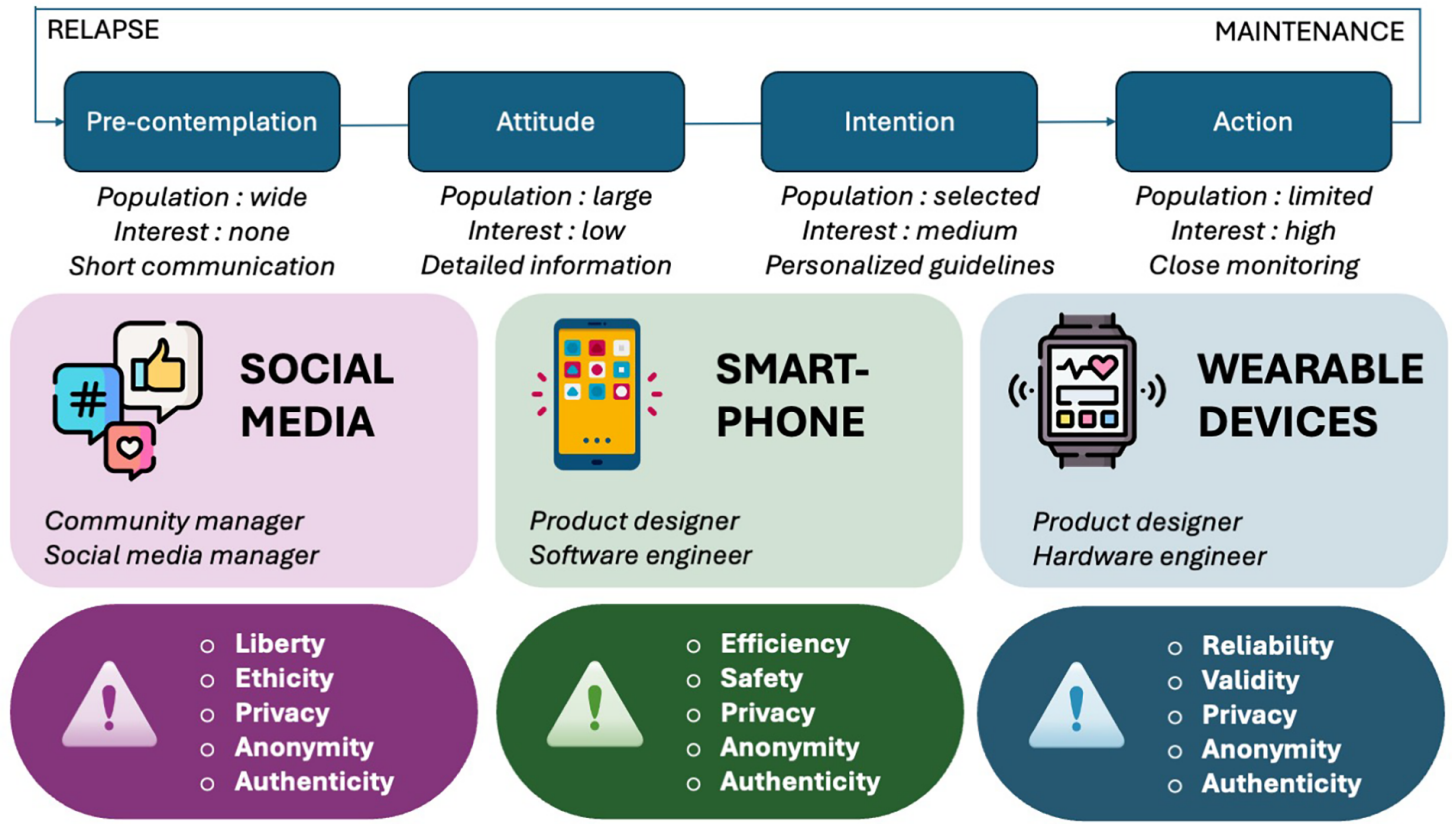
Summary of the roles, opportunities, and challenges associated with each new type of digital tool for mental health promotion.

The advent of digital tools offers unprecedented opportunities for mental health promotion, but it also introduces significant challenges. Social media platforms, chatbots, and wearables each play distinct roles in the enhancement of mental health by promoting health beliefs, sharpening intentions, and sustaining health behaviors. Social media can disseminate health information widely, fostering awareness and contemplation. Chatbots provide personalised, accessible support that can motivate behavioral changes and offer immediate assistance. Wearables offer real-time monitoring and feedback, aiding individuals in the maintenance of positive health behaviors through personalised insights and continuous engagement. When combined effectively, these new digital tools can transform our healthcare system towards accessible and comprehensive precision preventive medicine (Figure 1).

To maximize their potential, these new digital tools must be seamlessly integrated into clinical workflows. They can be positioned upstream or downstream, mandatory or optional, independently or in addition to mental healthcare providers (46, 47). However, they would probably need adequate psychoeducation programs on digital health literacy for both providers and users to integrate these technologies into their habits (48). Then, they would have the potential to become indispensable in addressing the numerous challenges faced by the traditional healthcare system, such as improving access to appropriate healthcare professionals (49), enhancing the quality of in-person consultations (48), and ultimately reducing overall healthcare costs (50, 51). Indeed, the financial sustainability of digital mental health interventions is an important factor in their widespread adoption. Several studies indicate that while the initial cost of developing and deploying digital tools (e.g., mobile applications, wearable devices) may be high, long-term benefits include reduced psychiatric hospital admissions, decreased reliance on medication, and fewer emergency mental health interventions (51). Systematic reviews of economic evaluations found that digital interventions can be cost-effective by improving early detection and intervention for mental health conditions (50). Governments and healthcare organizations should consider investing in these tools to promote long-term savings and improved patient outcomes.

Nevertheless, these technologies must be implemented thoughtfully to address potential issues such as privacy concerns, data security, and equitable access. Ensuring that digital health tools are inclusive and culturally sensitive is crucial for their effectiveness. Moreover, human interaction in mental health care should not be entirely replaced by technology; the integration of digital tools with conventional care models is essential for comprehensive support. In the realm of mental health promotion, special caution is necessary for patients with psychiatric disorders who may have impaired judgment, exposing them to a higher risk of misuse.

In these three situations (social media, chatbots and wearables), we suggested that:

- Privacy must be firmly protected,
- Anonymization should avoid risk of recognition and privacy violation when using social media platform,
- Each use should imply a consent from the user,
- One must keep in mind that LLM could potentially cause harm by perpetuating racist and gender ideas,
- The data collected (storage, sharing, ownership, dissemination) has to be used responsibly and under the rules ongoing in each country (when existing).

Generally speaking, concerning ethical issues in digital tool utilization to promote mental health, a new class of rights should arise to protect mental privacy, guard against algorithm bias; prevent personality-changing manipulations.

The rise of precision medicine based on technological tools also risks leading us towards a new model of health and well-being that may be disconnected from the patient's actual complaints and their physiological and medical reality. Healthcare providers must keep the patient and their concerns at the centre of care (52). In conclusion, the optimised and reasoned use of new digital tools in mental health can leverage technology to improve health outcomes, making mental health care more accessible, personalised, and effective in the digital era.
